# Glutamine Analogues Impair Cell Proliferation, the Intracellular Cycle and Metacyclogenesis in *Trypanosoma cruzi*

**DOI:** 10.3390/molecules25071628

**Published:** 2020-04-02

**Authors:** Rodolpho Ornitz Oliveira Souza, Marcell Crispim, Ariel Mariano Silber, Flávia Silva Damasceno

**Affiliations:** Laboratory of Biochemistry of Tryps–LaBTryps, Department of Parasitology, Institute of Biomedical Sciences, University of São Paulo, Av. Prof. Lineu Prestes 1374, 05508000 Sao Paulo, Brazil; rodolphosouza@usp.br (R.O.O.S.); marcell@usp.br (M.C.)

**Keywords:** Chagas disease, *Trypanosoma cruzi*, azaserine, acivicin, glutamine metabolism, cell death, differentiation

## Abstract

*Trypanosoma cruzi* is the aetiologic agent of Chagas disease, which affects people in the Americas and worldwide. The parasite has a complex life cycle that alternates among mammalian hosts and insect vectors. During its life cycle, *T. cruzi* passes through different environments and faces nutrient shortages. It has been established that amino acids, such as proline, histidine, alanine, and glutamate, are crucial to *T. cruzi* survival. Recently, we described that *T. cruzi* can biosynthesize glutamine from glutamate and/or obtain it from the extracellular environment, and the role of glutamine in energetic metabolism and metacyclogenesis was demonstrated. In this study, we analysed the effect of glutamine analogues on the parasite life cycle. Here, we show that glutamine analogues impair cell proliferation, the developmental cycle during the infection of mammalian host cells and metacyclogenesis. Taken together, these results show that glutamine is an important metabolite for *T. cruzi* survival and suggest that glutamine analogues can be used as scaffolds for the development of new trypanocidal drugs. These data also reinforce the supposition that glutamine metabolism is an unexplored possible therapeutic target.

## 1. Introduction

Chagas disease (CD) is caused by the flagellate protist *Trypanosoma cruzi* and affects approximately 8 million people worldwide [1], with some 25 million further people at risk of infection. It is endemic in the Americas where the vector transmission occurs [2]. However, cases of CD have been reported not only in the Americas but also in Europe, Asia and Oceania, mostly due to migration of infected people from endemic countries. Although the worldwide distribution, the CD treatment available nowadays is based in two drugs discovered many years ago: nifurtimox and beznidazole. The effect of these drugs is controversial, depending on the stage of disease, age and immune response of patient and the susceptibility of the *T. cruzi* genotype, moreover cause many side effects and the treatment required a prolonged course. [3,4,5,6].

*T. cruzi* has a complex life cycle, alternating between two hosts, a reduviid insect and many mammalian species, including humans. During its life cycle, this parasite differentiates several times into replicative and non-replicative forms. The insect vectors become infected during the blood meal, when they ingest the non-replicative, infective trypomastigotes present in the host bloodstream. In the insect midgut, the trypomastigotes differentiate into the replicative epimastigotes, which colonize the insect digestive tract. Once in the posterior part of the midgut, epimastigotes differentiate into infective, non-replicative metacyclic trypomastigotes, which are later expelled with faeces during the intake of the next blood meal. The infection of the mammalian host occurs when these parasites enter into contact with mucosae or wounds in the skin. Once inside the host cells, metacyclic trypomastigotes must infect the mammalian host cells to reach the host cell cytoplasm, where they differentiate into the replicative form, the amastigotes. After intracellular proliferation, amastigotes differentiate into trypomastigotes, passing through a transient form called the intracellular epimastigote. Trypomastigotes burst from host cells by lysis and can (i) invade neighbouring cells, colonizing the tissue; (ii) and reach the bloodstream, allowing the infection of other tissues; and (iii) can be ingested by a new triatomine insect during its blood meal [3,4,7,8].

During its life cycle, *T. cruzi* faces different physicochemical, chemical and nutritional conditions in each environment. In addition, the availability of energy and carbon sources is highly variable, contributing to the selection of parasites with remarkable metabolic flexibility (reviewed elsewhere [9]). For example, it is well documented that *T. cruzi* is able to consume glucose preferentially at the beginning of the exponential proliferation phase, and after exhaustion of this metabolite, they switch to a metabolism that is mainly based on the consumption of amino acids [10,11,12]. It is well known that a variety of amino acids are robust energy sources [13,14,15,16,17,18]. In addition, amino acids are important to crucial processes such as energy management [19], cell proliferation [20,21], cell differentiation [13,22,23,24], host cell infection [15], intracellular survival [23,25], osmotic control [26] and resistance to different types of stress conditions [14,27,28,29].

Glutamine (Gln) is a non-essential amino acid synthesized by most organisms. Its participation in nitrogen metabolism inside cells has been well documented, mainly in yeast [30] and cyanobacteria [31]. In humans, this amino acid is found at high concentrations in the plasma and in skeletal muscle. In cancer cells, Gln also has a large range of functions, acting as an ATP and carbon source for the biosynthesis of lipids and stimulating cell proliferation, enhancing the redox potential and serving as a substrate for nucleotide biosynthesis [32].

Gln can be internalized from the external medium and/or synthesized through the enzyme glutamine synthetase (GS), which uses ammonium, ATP and glutamate (Glu) as substrates. In turn, Gln is used as an amide- or amine-group donor or as a substrate, being a true metabolic hub for the distribution of -NH_2_ groups through various biochemical pathways, such as those for amino sugar, guanosine, or pyrimidine biosynthesis. [32,33,34,35]. Recently, we demonstrated that *T. cruzi* is able to take up Gln from external medium [13] and synthesize it using a canonical biosynthesis pathway [36]. In those studies, we showed that the biosynthesis and uptake of Gln are developmentally regulated according to Gln availability in the extracellular environment and cell demand. We also showed that Gln biosynthesis participates in ammonium detoxification, which is critical for the parasite to escape the parasitophorous vacuole during host-cell infection [13,36]. 

**Figure 1:**
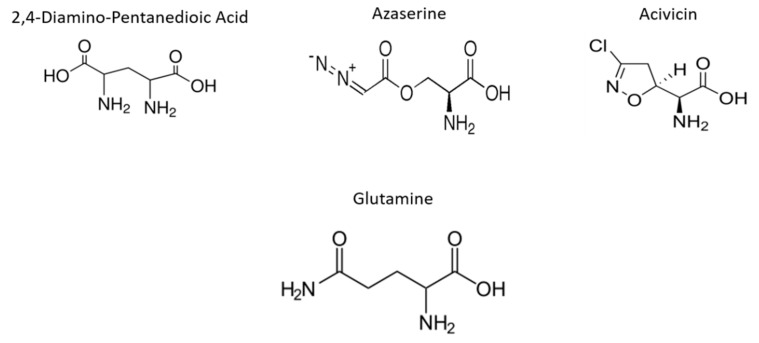
Structure of Gln and its analogues. Gln analogues used to evaluate its interference in the biological process during the *T. cruzi* life cycle.

In the present study, we evaluated the impact of the Gln analogues acivicin (ACV), azaserine (AZA) and 2,4-diaminopentanedioic acid (DPA, Figure 1) on the cell biology and life cycle of *T. cruzi* according to the hypothesis that they will preferentially interfere with enzymes that recognize Gln as a substrate, product or allosteric modulator. Our results showed that these Gln analogues impair parasite proliferation and differentiation and affect the intracellular cycle and metacyclogenesis. Our data demonstrated that Gln metabolism in *T. cruzi* can be explored as therapeutic target.

## 2. Results

### 2.1. Gln analogues Impair the Proliferation of the Epimastigote Form

**Figure 2:**
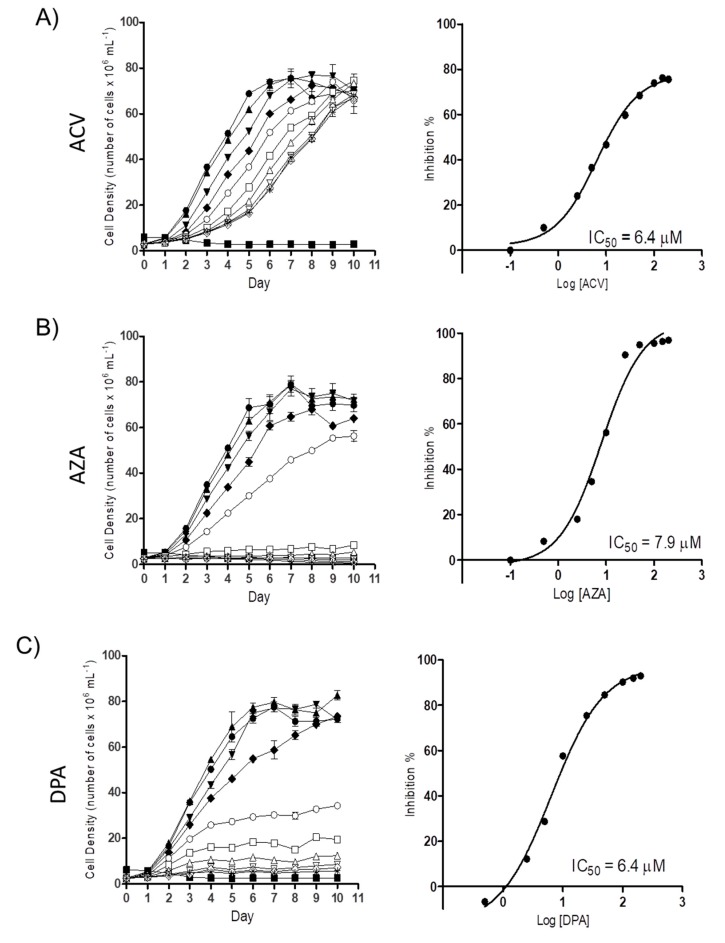
The effect of the Gln analogues on the proliferation of the epimastigote form. Left panels: Epimastigote proliferation in LIT medium in the presence of ACV (**A**), AZA (**B**) or DPA (**C**) (from 0.5 µM to 200 µM) was observed for as many as ten days. Symbols: black circle: 0 µM; black up-pointing triangle: 0.5 µM; black down-pointing triangle: 2.5 µM; black diamond: 5 µM; white circle: 10 µM; white square: 25 µM; white up-pointing triangle: 50 µM; white down-pointing triangle: 100 µM; white diamond: 150 µM; and star: 200 µM. Inhibition control: 60 µM rotenone and 0.5 µM antimycin. Right panels: dose response curves derived from the proliferation curves (data from the 5th day) when the parasites were in the mid-exponential growth phase. Data were processed and plotted using the GraphPad Prism 6.1 software program. The assay was performed in biological triplicates.

To determine the possible trypanocidal or trypanostatic effect of the Gln analogues on *T. cruzi*, we initially evaluated their effect on epimastigote proliferation. Proliferating parasites were treated with different concentrations of each Gln analogue (5 µM to 200 µM), and their cell densities were observed for as many as 10 days. All the analogues showed a dose-dependent effect on parasite proliferation. At the time corresponding to the mid-exponential growth phase (5th day of proliferation), the concentration corresponding to 50% inhibition (IC_50_) was calculated, with values of 6.2 ± 1.01 µM, 6.1 ± 2.1 µM and 6.2 ± 0.72 µM for ACV, AZA and DPA, respectively (Figure 2). These results showed that all three analogues had relevant activity against parasite proliferation at low micromolar range.

### 2.2. Gln analogue ACV Induces Extracellular Phosphatidylserine Exposure

**Figure 3:**
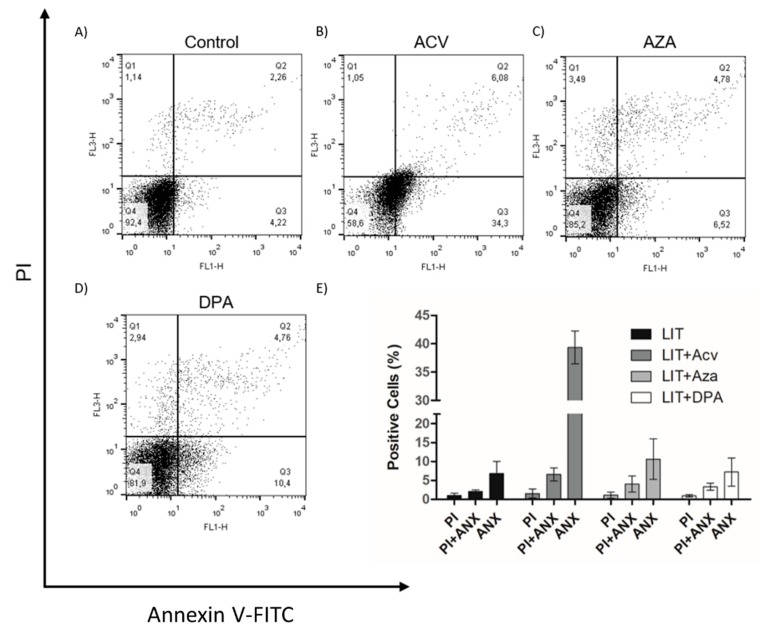
The effect of Gln analogues on the extracellular phosphatidylserine exposure and membrane integrity. Parasites in the exponential growth phase were treated with Gln analogues (IC_50_ value) for 48 h. After incubation, the parasites were labelled with propidium iodide (PI) and annexin V-FITC (ANX) and analysed by flow cytometry; (**A)** Control, (**B)** ACV, (**C)** AZA, (**D)** DPA. (**E)** Bars correspond to three independent mean values from three independent experiments. Error bars represent the standard deviation among biological replicates.

Once Gln analogues impaired cell proliferation, we evaluated whether they induced programmed cell death (PCD) or necrosis in the parasites. PCD is a process characterized by morphological, biochemical, and cellular events, such as extracellular phosphatidylserine exposure, DNA fragmentation, decreased ATP levels, and increased levels of ROS, Ca^2+^ and cytochrome c release [37,38]. To analyse whether the treatments of cells with the Gln analogues induced PCD, we treated epimastigotes with concentrations of the analogues equivalent to the IC_50_ value of each for 48 h. After the treatments, the parasites were labelled with propidium iodide (PI) to analyse membrane integrity and annexin V-FITC to evaluate phosphatidylserine exposure (PS) as determined by flow cytometry (Figure 3). Although all Gln analogues impaired cell proliferation, only ACV at a concentration corresponding to the IC_50_ induced PS exposure in approximately 34% of the cells. AZA and DPA at concentrations corresponding to their respective IC_50_ had no detectable effect on PS exposure. Under all conditions, the parasites maintained membrane integrity similar to that of the control. These data suggest that treatment with ACV affects parasite metabolism, triggering PCD, whereas AZA and DPA likely had only a trypanostatic effect.

### 2.3. Recovery of Parasites after Treatment with Gln analogues

**Figure 4:**
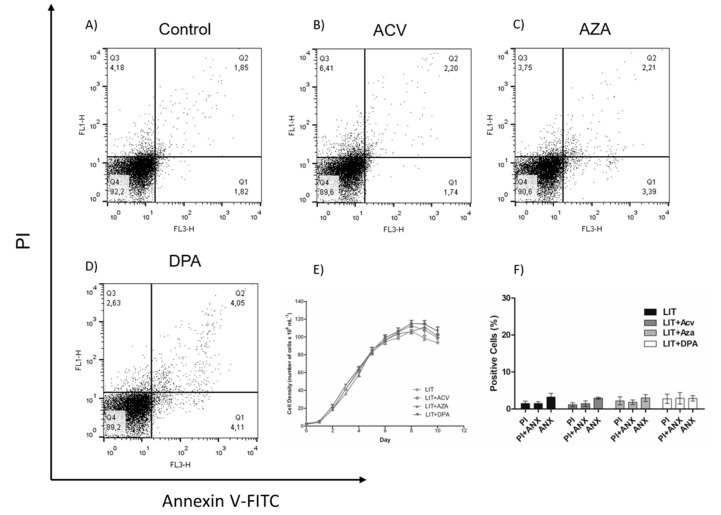
Recovery of the epimastigotes after Gln analogues treatment. The parasites were treated for 48 h with Gln analogues (IC_50_ value concentration). Then, the treatment was removed, and the parasites were cultivated in LIT medium. Proliferation was observed for as many as 10 days (**E**). On the fifth day, the parasites were labelled with propidium iodide (PI) and annexin V-FITC to evaluate cell death by flow cytometry: (**A**) control, (**B**) ACV, (**C**) AZA and (**D**) DPA. (**F**) Bars correspond to three independent mean values from three independent experiments. Error bars represent the standard deviation among biological replicates.

To evaluate whether the effect of the Gln analogues is reversible, epimastigotes were treated for 48 h with Gln analogues (concentrations corresponding to their IC_50_ values). Then, the compounds were washed away, and the parasites were maintained in LIT medium without Gln analogues. Parasite proliferation was observed for as many as ten days to analyse the recovery of their proliferative profile (Figure 4E). We also evaluated parasite cell death on the fifth day of recovery. To make these observations, the parasites were labelled with PI and annexin V-FITC (Figure 4A–D). Surprisingly, the obtained data showed that, in the case of ACV-induced membrane PS exposure, the PS exposure was reversed, and in all cases, the proliferation was recovered to the expected level when the treatments were removed. These data show that the Gln analogue effect (at the used concentrations) is trypanostatic rather than trypanocidal.

### 2.4. Effect of Gln analogues on Metacyclogenesis

**Figure 5:**
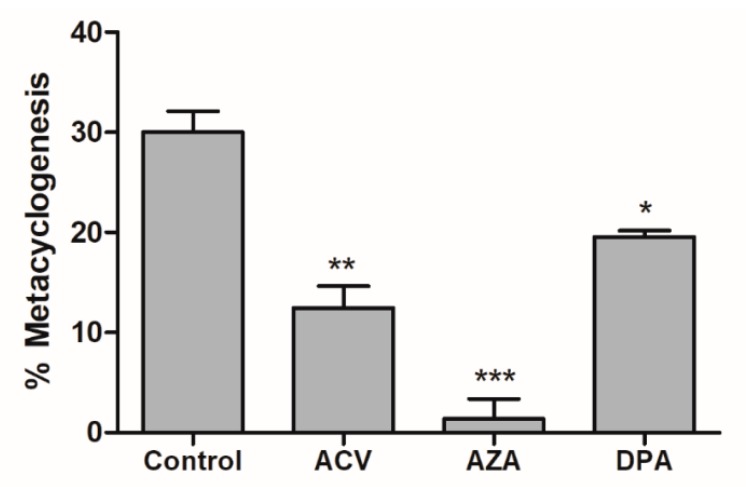
Effect of Gln analogues on the metacyclogenesis process. The epimastigote parasites (stationary growth phase) were maintained in TAU Gln medium in the presence of each Gln analogue (IC_50_ value concentrations) or not (control). The differentiation was evaluated by counting in a Neubauer chamber on the 6th day. This experiment was performed in triplicate. Statistical analysis: One-way ANOVA followed by Tukey post-test; *p* < 0.05, using the GraphPad Prism 6.1 software program. The assays were performed in biological triplicates and error bars represent the standard deviation. We represent in this figure the level of statistical significance as follow: *** *p* value < 0.001; ** *p* value < 0.01; * *p* value < 0.05.

Metacyclogenesis is a crucial differentiation process involving intensive cell remodelling. This process can be performed in vitro by submitting epimastigotes to controlled (2 h) metabolic stress in TAU medium and then supplying carbon and energy sources. Contreras et al. showed that a combination of glucose, aspartate, glutamate and proline trigger and support this differentiation [22]; more recently, it was shown that Gln by itself is able to support metacyclogenesis to levels comparable to those of parasites in standard medium (TAU 3AAG) [13]. To evaluate the effect of the Gln analogues on the metacyclogenesis promoted by Gln, parasites in the epimastigote form in the stationary growth phase were induced to differentiation in TAU-Gln in the presence of each Gln analogue (IC_50_ value). The obtained data showed that the Gln analogues acted as metacyclogenesis inhibitors, decreasing the differentiation from the epimastigote to the metacyclic trypomastigote form. The inhibition was approximately 59%, 90% and 35% for ACV, AZA and DPA, respectively (Figure 5).

### 2.5. Effect of the Gln analogues on the Intracellular Infection of T. cruzi

**Figure 6:**
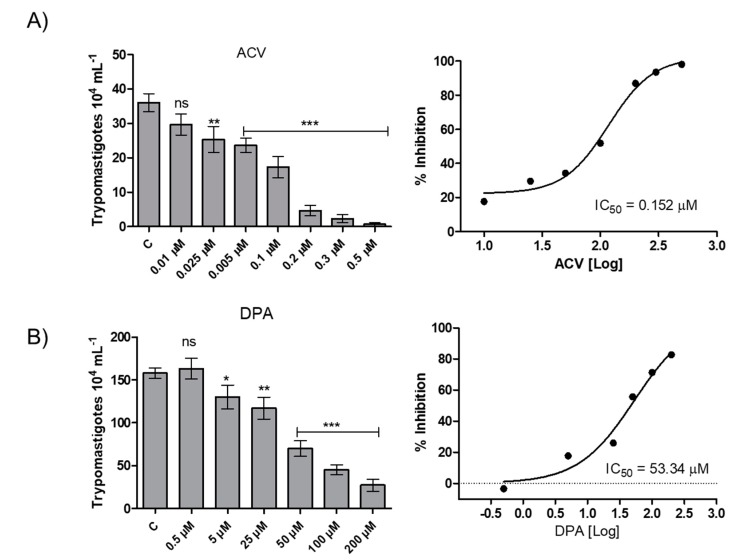
Effect of the Gln analogues on the intracellular cycle of *T. cruzi*. CHO-K_1_-infected cells were treated with different concentrations of each Gln analogue. On the fifth day post-infection, the burst trypomastigotes were counted in a Neubauer chamber. (**A**) ACV, (**B**) DPA. The IC_50_ value was calculated using a classical sigmoidal dose-response curve, and the statistical analysis was performed with one-way ANOVA followed by Tukey post-test *p* < 0.05, using the GraphPad Prism 6.1 software program. We represent in this figure the level of statistical significance as follow: *** *p* value < 0.001; ** *p* value < 0.01; * *p* value < 0.05. For *p* value > 0.05 we consider the differences not significant (ns).

To evaluate the effect of the Gln analogues on the infection in mammalian host cells, we initially evaluated the analogues toxicity in CHO-K_1_ cells, the cell system chosen as the parasitic host cell. The AZA and ACV analogues were toxic at concentrations above 0.5 µM, whereas the DPA analogue had no toxic effect at concentrations up to 200 µM (Figure S1). Then, CHO-K_1_ cells were infected with trypomastigotes and treated with Gln analogues at concentrations up to their toxicity limit, and the number of trypomastigotes released into the culture medium from the infected cells was quantified. AZA at a concentration of 0.5 µM inhibited 20% of trypomastigote burst (Figure S2). ACV and DPA showed a dose-dependent inhibition of trypomastigote burst in the infected cells, with IC_50_ values of 0.152 µM and 53.3 µM, respectively (Figure 6). Given that DPA has a relevant effect on intracellular parasites at a high concentration, our data re-establish interest in the use of ACV as a potential starting compound for developing a trypanocidal agent against *T. cruzi*.

## 3. Discussion

**Figure 7:**
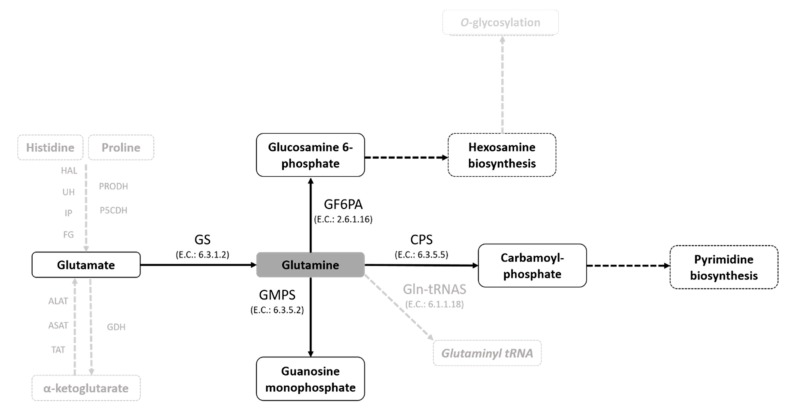
Glutamine-dependent metabolic pathways in *T. cruzi*. A schematic view of Gln metabolism in *T. cruzi* based on the gene sequences annotated in genome databases (TriTrypDB and KEGG pathway databases).

In this study, we demonstrate the effect of the Gln analogues ACV, AZA and DPA on the life cycle of *T. cruzi*. We had previously shown that *T. cruzi* can take up Gln and/or engage in biosynthesis in all stages of the *T. cruzi* life cycle, except for the bloodstream trypomastigotes. We also demonstrated that Gln supports the differentiation from the epimastigote to the metacyclic trypomastigote form, maintaining cell viability during metacyclogenesis and participating in energy metabolism [13,36]. In addition, in *T. cruzi,* Gln can be the substrate for extremely important metabolic pathways of cell survival (Figure 7).

The Gln analogues used in this study did not impair Gln uptake [13] or the *T. cruzi* GS activity (Figure S3). However, all of them inhibited the *T. cruzi* enzyme glutamine fructose-6-phosphate aminotransferase (GF6PA) activity (Figure S4). GF6PA is an enzyme that utilizes Gln as an amino donor for glucosamine biosynthesis and is the first step in the hexosamine biosynthetic pathway (HBP). Particularly, AZA has been described as an inhibitor of HBP, and its effects are related to glucose homeostasis and glycosylation of proteins associated with insulin resistance in type II diabetes [39,40]. In trypanosomatids, AZA was studied many years ago mainly to assess its action as an inhibitor of nucleic acid metabolism, with the findings demonstrating that AZA does not affect purine or pyrimidine biosynthesis [41]. In early infections in mice with *Trypanosoma equiperdum*, treatment with AZA diminished the number of parasites [42]. In this parasite, the analogue inhibits the incorporation of sugar nucleotides, impairing nucleic acid metabolism. The same study showed that Gln and other amino acids diminish the effects of AZA [43]. Interestingly, the findings reported herein on the inhibition of HBP by AZA are in agreement with the absence of effects previously reported for *T. cruzi* nucleic acid metabolism.

The analogue DPA also had an anti-proliferative effect on the epimastigote forms, inhibiting GF6PA activity and impairing the intracellular cycle of the parasite. Moreover, DPA was well tolerated by the host cell compared with other analogues studied in this study (four hundred-fold less toxicity). Interestingly, we did not find any description in the literature about the mechanism of action for this compound, and to our knowledge, this is the first study to report the possible cell targets for this Gln analogue.

ACV is a Gln analogue produced by fermentation of *Streptomyces sviceus* and was previously studied as a potential anticancer drug [44]. The best-known mechanism of action depends on the inhibition of CTP synthetase, an enzyme critical for the biosynthesis of sugar nucleotides. Many side effects were reported from the clinical trials of ACV as an anticancer drug and are explained by several instances of off-targeting inside the cell. ACV is an inhibitor of not only Gln-dependent enzymes but also enzymes with aldehyde dehydrogenase activity [45]. When studied in trypanosomatids, this Gln analogue was found to act as an inhibitor of CTPs in *T. brucei*, and was thus proposed as a compound useful for guiding the synthesis of new anti-*T. brucei* drugs [46]. Additionally, for *T. brucei*, ACV was described as an inhibitor of GMP synthase, another enzyme that utilizes Gln as an amide donor [47]. Our results showed that ACV is a *T. cruzi* proliferation inhibitor, specifically of those in the epimastigote form, in a dose-dependent manner. In addition, this analogue induced extracellular phosphatidylserine exposure, indicating that the PCD process was triggered. PCD in trypanosomatids is a well-characterized process, and parasites can trigger PCD after drug treatment or cultivation [48,49,50]. Interestingly, in this case, the effect of phosphatidylserine exposure was reversed after the ACV treatment was removed. ACV has also been shown to be effective in the intracellular cycle, diminishing the number of burst trypomastigotes in a dose-dependent response at concentrations in the nanomolar range. Importantly, ACV inhibits *Tc*GF6PA activity, making *Tc*GF6PA a putative new target for ACV. These data suggest that, as described for *T. brucei*, ACV can be used as a scaffold compound to guide the synthesis of new molecules against *T. cruzi* with few or no side effects in the host.

Finally, we demonstrated that Gln analogues impair the differentiation from the epimastigote to the metacyclic trypomastigote form that had been promoted by Gln, reinforcing the importance of this amino acid in the metacyclogenesis process. Taken together, these results showed the importance of Gln for *T. cruzi* survival. Gln analogues impaired important biological processes, such as cell proliferation, differentiation and the intracellular cycle.

## 4. Materials and Methods

### 4.1. Parasites and Mammalian Cells

The epimastigote form of the *T. cruzi* CL strain and 14 clones were maintained in the exponential phase by sub-culturing every 48 h in liver infusion tryptose (LIT) medium supplemented with heat-inactivated FCS (foetal calf serum) 10% at 28 °C [51]. Chinese hamster ovary cells (CHO-K_1_ cells) were maintained in RPMI medium supplemented with FCS 10%, 0.15% (w/v) NaCO_3_, 100 units·mL^−1^ penicillin and 100 g·mL^−^¹ streptomycin in a humidified atmosphere with 5% CO_2_ [23].

### 4.2. Proliferation Inhibition Assays

*T. cruzi* epimastigote forms in the exponential growth phase (4.0–6.0 × 10^7^ mL^−1^) were treated with different concentrations of each analogue: ACV, AZA and DPA (purchased from Sigma-Aldrich, San Luis, MO, USA) or not treated (negative control) in LIT medium. As a positive control, we used a combination of rotenone (60 µM) and antimycin (0.5 µM) [28]. The parasites (2.5 × 10^6^ mL^−1^) were transferred to 96-well plates and incubated at 28 °C. Quantification of cell proliferation was performed by reading the optical density (OD) at λ 620 nm for eight days. The OD values were converted to cell numbers using a linear regression equation previously obtained under the same conditions. The IC_50_ was calculated by using the cell numbers obtained in the exponential growth phase (5th day of proliferation). To determine the IC_50_ value, the obtained values were fitted to a nonlinear regression function: a sigmoidal dose-response curve [28,48]. Each compound was evaluated in biological triplicates with technical quadruplicates in each experiment.

### 4.3. Extracellular Phosphatidylserine Exposure Analysis

Epimastigotes in the exponential growth phase were maintained in LIT and treated with Gln analogues (concentrations corresponding to the IC_50_) for 48 h. Then, the parasites were washed in annexin buffer (10 mM HEPES, 140 mM NaCl, and 5 mM CaCl_2_, pH 7.4) and resuspended in 50 µL of annexin buffer. The cells were incubated with a mixture of propidium iodide (1 µg·µL^−1^) and annexin (1 µM) for 15 min at room temperature. After incubation, the reaction was stopped by the addition of annexin buffer (450 µL), and the cells were analysed by flow cytometry (FACSCalibur, BD Biosciences, Franklin Lakes, NJ, USA) as previously described [48].

### 4.4. Effect of the Gln analogues on Metacyclogenesis

Epimastigotes were maintained in LIT medium until reaching the stationary phase (~1.0 × 10^8^ cells·mL^−1^), washed twice with PBS, resuspended in triatomine artificial urine (TAU) medium (190 mM NaCl, 17 mM KCl, 2 mM MgCl_2_, 2 mM CaCl_2_, and 8 mM potassium phosphate buffer, pH 6.0) [22], and incubated for 2 h at 28 °C. Then, the cells were centrifuged (5 min, 1600× *g* at room temperature) and resuspended in TAU Gln (190 mM NaCl, 17 mM KCl, 2 mM MgCl_2_, 2 mM CaCl_2_, 8 mM potassium phosphate buffer, and 10 mM Gln, pH 6.0) [13] in the presence or absence (control) of each Gln analogue: ACV, AZA or DPA (concentration corresponding to the IC_50_). On the 6^th^ day, after triggering metacyclogenesis, the parasites were counted in a Neubauer chamber, and the number and percentage of the MTs were calculated.

### 4.5. Effect of the Gln analogues on Mammalian Cell Viability

Chinese hamster ovary cell line (CHO-K_1_) cells (5.0 × 10^5^ per well) were maintained in 24-well plates in RPMI medium supplemented with FCS (10% *v*/*v*) at 37 °C and 5% CO_2_ in the presence or absence (control) of different concentrations of each Gln analogue for 48 h. After the incubation time, cell viability was evaluated by MTT assay [52]. The cells were washed twice in PBS, and PBS (200 µl) and MTT (0.25 mg/mL) were added to each well. The cells were incubated for 3 h (37 °C in 5% CO_2_), and the reaction was stopped by the addition of SDS (200 µL of 10% SDS per well). The cells were lysed by up and down pipetting. The viability (colorimetric reaction) was measured by spectrophotometry at 595–690 nm in a SpectraMax i3 spectrophotometer (Molecular Devices, San Jose, CA, USA).

### 4.6. Effect of the Gln analogues on Trypomastigote Bursting

CHO-K_1_ cells (5.0 × 10^4^ per well) were maintained in 24-well plates as described previously. Briefly, the cells were infected with the trypomastigote form (2.5 × 10^6^ trypomastigotes per well) for 3 h. Then, the parasites remaining in the supernatant were removed, the cells were washed twice with PBS, and RPMI medium was added to the culture. The infected cells were treated with different concentrations of each Gln analogue and kept in an incubator (37 °C with a 5% CO_2_ humid atmosphere). On the fifth day after infection, the trypomastigotes present in the supernatant were counted in a Neubauer chamber.

## Supplementary Materials

The following are available online: Figure S1: Viability of CHO-K_1_ cells treated with different concentrations of ACV and DPA. Figure S2: Effect of AZA on cell viability and trypomastigote burst. Figure S3: Effects of Gln analogues on GS activity in the extracts from the epimastigotes. Figure S4: Effects of Gln analogues on GF6PA activity in the extracts from the epimastigotes.
