# Supplementary material for: Glutamine Analogues Impair Cell Proliferation, the Intracellular Cycle and Metacyclogenesis in Trypanosoma cruzi
Source: Molecules. 2020 Apr 2;25(7):1628. doi: 10.3390/molecules25071628 (PMC7180609; doi:10.3390/molecules25071628)
Supplement: Supplementary file 1 [file molecules-25-01628-s001.pdf]

Supplementary Materials: The following are available online: Fig. S1: Viability of CHO-K_1 cells treated with different concentrations of ACV and DPA. Fig. S2: Effect of AZA on cell viability and trypomastigote burst. Fig. S3: Effects of Gln analogues on GS activity in the extracts from the epimastigotes. Fig. S4: Effects of Gln analogues on GF6PA activity in the extracts from the epimastigotes.

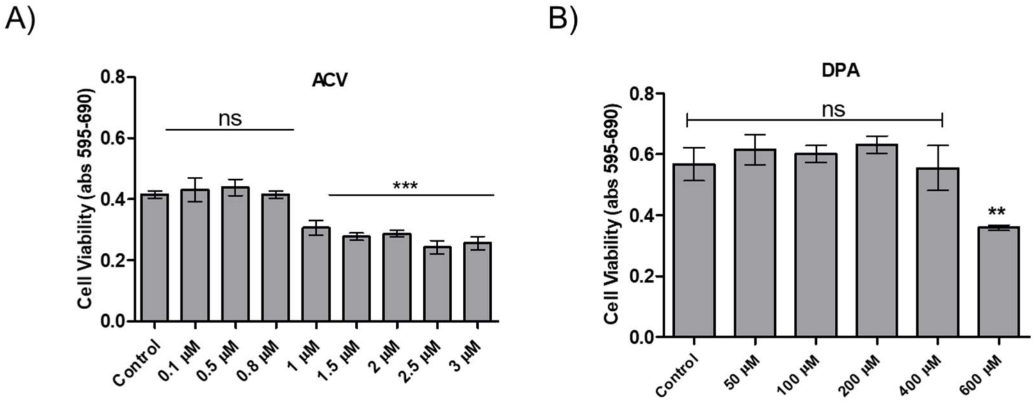

Figure S1: Viability of CHO-K_1 cells treated with different concentrations of ACV and DPA. A) ACV and B) DPA. The cells were treated with different concentrations of ACV (from 0.1 µM to 3 µM) and DPA (from 50 µM to 60 µM). Cell viability was assessed by MTT assay after 48 h of incubation. The assays were performed in biological triplicates and error bars means standard deviation among biological replicates. We represent in this figure the level of statistical significance as follow: *** p value <0.001; ** p value <0.01; * p value <0.05. For p value >0.05 we consider the differences not significant (ns).

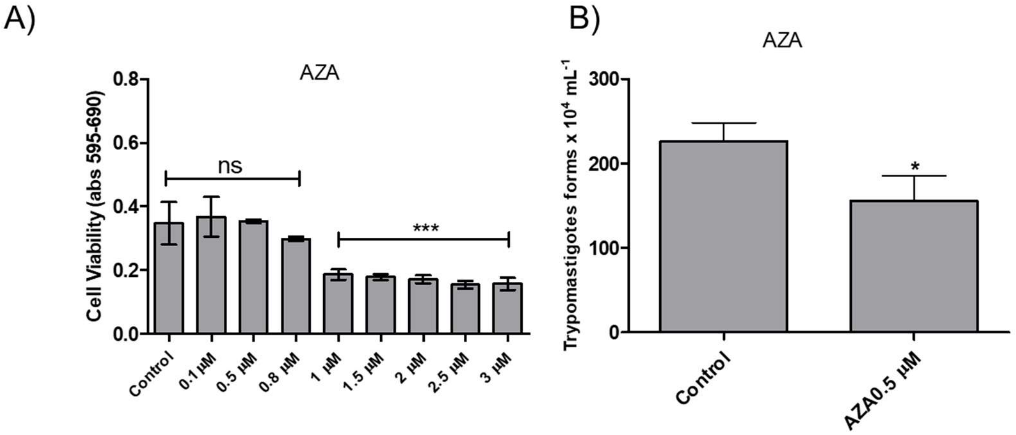

Figure S2: Effect of AZA on cell viability and trypomastigote burst. (A) CHO-K_1 cells were incubated for 48 h in the presence of different concentrations of AZA (from 0.1 µM to 3 µM). Cell viability was assessed by MTT assay. (B) CHO-K_1 cells were infected with *T. cruzi* (trypomastigote form) and treated with AZA (0.5 µM). The number of trypomastigotes that burst in infected cells on the 5th day post-infection was counted in a Neubauer chamber. The assays were performed in biological triplicates and error bars means standard deviation among biological replicates. We represent in

this figure the level of statistical significance as follow: *** p value <0.001; * p value <0.05. For p value >0.05 we consider the differences not significant (ns).

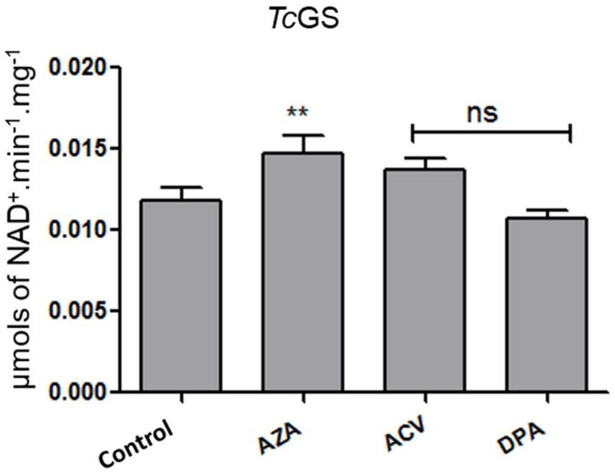

Figure S3: Effects of Gln analogues on GS activity in the extracts of the epimastigotes. The enzyme activity was evaluated in the presence of each Gln analogue (100 µM) for 5 min by monitoring the absorbance of NADH oxidation at 340 nm in a spectrophotometer as described by Crispim et al. 2018 [36]. The experiment was performed in biological triplicate and analysed by one-way ANOVA followed by Tukey post-test; $p < 0.05$. The assays were performed in biological triplicates and error bars means standard deviation among biological replicates. We represent in this figure the level of statistical significance as follow: ** p value <0.01; for p value >0.05 we consider the differences not significant (ns).

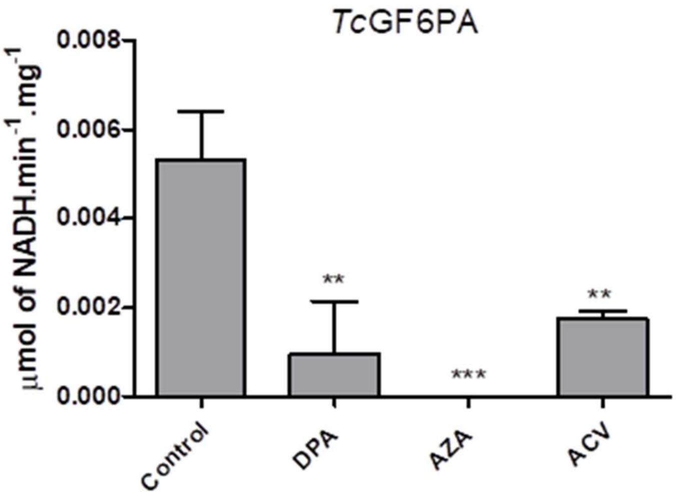

Figure S4: Effects of Gln analogues on **GF6PA** activity in the extracts of the epimastigotes. The GF6PA activity was measured with a two-step reaction: in the first step, the crude extracts of epimastigotes were

incubated in the presence of 50 mM Tris HCL, 1 mM Gln, 1.5 mM fructose-6-phosphate, and 1 mM EDTA (the substrates of the GF6PA enzyme) in the presence or absence of each Gln analogue (100 μM) for 5 min. The reaction was stopped by the addition of trichloroacetic acid (TCA). In the second step, Glu (the product of GF6PA reaction) was quantified according to the activity of the enzyme glutamate dehydrogenase (Sigma-Aldrich) by monitoring the NAD^+ reduction at λ 320 nm. The resulting NADH value was quantified. The experiment was performed in biological triplicate and analysed by one-way ANOVA followed by Tukey post-test; $p < 0.05$. The assays were performed in biological triplicates and error bars means standard deviation among biological replicates. *** p value <0.001; ** p value <0.01.
